# Adjuvants in the Driver’s Seat: How Magnitude, Type, Fine Specificity and Longevity of Immune Responses Are Driven by Distinct Classes of Immune Potentiators

**DOI:** 10.3390/vaccines2020252

**Published:** 2014-04-10

**Authors:** Elke S. Bergmann-Leitner, Wolfgang W. Leitner

**Affiliations:** 1 US Military Malaria Research Program, Malaria Vaccine Branch, 503 Robert Grant Ave, 3W65, Silver Spring, MD 20910, USA; 2 Division on Allergy, Immunology and Transplantation, National Institute of Allergy and Infectious Diseases, National Institutes of Health, 6610 Rockledge Drive, Bethesda, MD 20892, USA; E-Mail: wleitner@niaid.nih.gov

**Keywords:** vaccine, adjuvant, infectious disease, immune epitope, immune mechanism, Th1, Th2, Th17, mucosal immunity

## Abstract

The mechanism by which vaccine adjuvants enhance immune responses has historically been considered to be the creation of an antigen depot. From here, the antigen is slowly released and provided to immune cells over an extended period of time. This “depot” was formed by associating the antigen with substances able to persist at the injection site, such as aluminum salts or emulsions. The identification of Pathogen-Associated Molecular Patterns (PAMPs) has greatly advanced our understanding of how adjuvants work beyond the simple concept of extended antigen release and has accelerated the development of novel adjuvants. This review focuses on the mode of action of different adjuvant classes in regards to the stimulation of specific immune cell subsets, the biasing of immune responses towards cellular or humoral immune response, the ability to mediate epitope spreading and the induction of persistent immunological memory. A better understanding of how particular adjuvants mediate their biological effects will eventually allow them to be selected for specific vaccines in a targeted and rational manner.

## 1. The Advent of Adjuvants—A Brief History

Vaccines have gone through a dramatic evolution over the last century and although a large number of routinely used vaccines are relatively “old” (*i.e.*, are based on traditional approaches to vaccination), many new approaches and technologies are in the pipeline (reviewed in [1]). Traditional vaccines use the entire pathogenic organism to induce a protective immune response against disease. Various approaches are employed to remove the organism’s virulence and pathogenicity during vaccine manufacturing. Some of these processes are crude and unpredictable such as radiation or continued passaging *in vitro*, while newer approaches are highly targeted such as the selective disabling of genes associated with pathogenesis and/or survival of the pathogen. The risk of inadvertent infection due to insufficient attenuation of the pathogenic organism is overcome by harsh attenuation procedures such as treatment of the pathogen with formaldehyde or complete disruption of the pathogenic organism (e.g., detergent-split flu vaccine [2]). However, not all pathogens can be converted into effective vaccines by attenuation. An example is the attempt since the 1960s to use malaria parasites as vaccine by immunizing with sporozoites attenuated through irradiation [3], or the repeated *in vivo* attenuation of the infectious malaria parasite through drug treatment following infection which is designed to convert the pathogen into a vaccine inside the host rather than *in vitro*. These approaches only result in highly strain-specific (and relatively short-term) immunity, which is of no value in the field and of only limited value for travelers or military personnel [4].

Traditional vaccines have had an enormous impact on human health. They have resulted in the eradication (smallpox), almost complete elimination (polio), or a dramatic reduction in number of cases worldwide (measles, mumps, tetanus, whooping cough). Nevertheless, the need for new vaccines remains high. Novel vaccines are needed not only for diseases for which no or insufficiently effective vaccines exist (HIV, malaria, TB), but also to replace currently available vaccines with vaccines that are less reactogenic and safer (e.g., the RotaShield^®^ vaccine against rotavirus was withdrawn in 1999 because of several high-profile serious adverse events [5]). In addition, it would be advantageous to have (a) vaccines that require fewer immunizations, which increases compliance with vaccination regimens and thus improves the overall effectiveness of the vaccine in the population, and (b) vaccine which are effective in special populations such as newborn children and two steadily growing groups in the developed world: the elderly and immune-compromised individuals. To achieve a high level of safety and efficacy, many newer vaccines rely on potent immune-stimulators: vaccine adjuvants. Numerous reviews have been written about adjuvants, describing, for example, the immunological activity of various compounds [6], clinical adjuvants [7], adjuvants for mucosal vaccines [8], genetic adjuvants for DNA vaccines [9], or adjuvants for vaccines against select diseases such as influenza [10,11]. The present review will look at this booming research area from a different angle. We will discuss adjuvants in terms of their impact on the type of adaptive immune response that is generated and review contentious aspects of the mechanism of action of frequently used adjuvants.

### What Is an Adjuvant? The Futile Attempt to Categorize

Vaccine adjuvants are defined by the effect they have on innate and adaptive immune responses rather than their molecular structure or origin. They are highly diverse with no common structural or chemical features; they come from a wide range of sources and exhibit their immune-enhancing/immune-skewing activity through a broad variety of molecular and cellular mechanisms. Not only “immune potentiators” such as Pathogen Associated Molecular Patterns (PAMPs)—or their synthetic derivatives or the increasing number of small-molecule agonists which mimic their activity—but also particulate antigen-delivery systems are capable of initiating and/or enhancing immune responses. Thus, by definition, both are classified as adjuvants. Clearly separating them is impossible. Delivery systems not only aggregate antigens, protect them from rapid degradation, stabilize a protein’s structure, and provide a depot effect, but often also activate innate immune responses and thus have dual function. Such delivery systems can be further “enhanced” by combining them with PAMPs to either boost or (re)-direct immune responses and thus not only enhance the intensity of the adaptive response, but also its quality and type such as the T helper (Th)1 *vs.* Th2 bias. In conclusion, an adjuvant is any substance, compound or even strategy which results in the enhancement of adaptive immune responses when delivered together with an antigen.

## 2. Why Use Adjuvants? The Fundamental Rationale and How It Has Changed over Time

Very few antigens are inherently immunogenic and virtually all vaccines require adjuvants in some form, endogenous or exogenous. Without a component that engages either innate immune cells or additional receptors on lymphocytes such as complement receptors [12], most non-adjuvanted, highly-purified antigens induce tolerance rather than immunity [13]. Very few antigens, such as certain toxins, are capable of inducing antibody responses when administered without adjuvants. Because of their immunogenicity, non-toxic derivatives of some toxins are being developed as adjuvants themselves, such as cholera toxin (CT) or *E. coli* enterotoxin (LT) (reviewed in [14]). The first scientific reports of exogenous adjuvants deliberately added to vaccines are less than a century old and come from Gaston Ramon in the 1920s [15]. The substances he added to vaccines to “enhance immune responses” were complex and poorly defined and included tapioca starch and agarose. These early adjuvants, however, did trigger inflammation, which subsequently enhanced vaccine-specific lymphocyte responses. Adjuvanticity in this scenario is through a bystander effect with a significant amount of “wasted inflammation” (Quote from N.M. Valiante (Novartis Vaccines)), defined as excessive innate immune responses, which result in reactogenicity but only partially contribute to the adaptive immune response. To this day, the production and release of innate immune factors (such as inflammatory cytokines) is frequently used to judge the “potency” of a vaccine adjuvant. While this can be a useful tool to identify novel candidates, the intensity of the inflammatory response does not necessarily correlate with the usefulness of an innate immune stimulator as a vaccine adjuvant. 

As a result of the elimination of many deadly or debilitating diseases through vaccination, public awareness of these diseases’ impact on society has vanished. Instead, the discussion has shifted from the benefit to the comparatively negligible risk of vaccination. Expectations regarding the safety and tolerability of preventive vaccines continue to increase, driving the development of novel adjuvants and adjuvanting strategies that decrease the amount of local inflammation and, ideally, eliminate any systemic innate immune activation, but without compromising the adjuvant effect. Modern adjuvants and innovative vaccine formulations are making it possible to dissociate strong inflammation from strong adjuvanticity. This provides a potent adjuvant effect in the absence of significant or deleterious inflammation, such as peptide-based nanofibers [16], nanoparticles [17] or mucosally-delivered nanoemulsions [18].

### Attenuation and Its Impact on the Immune Response

Improving the safety of whole-organism-based vaccines by increasing the level of attenuation is almost inevitably associated not only with lower immunogenicity, but also a significant change in the type of the immune response these vaccines induce. Fully attenuated (“dead”), disrupted (e.g., by detergent) or subunit vaccines (e.g., recombinant proteins) are primarily routed through the MHC-II processing pathway of antigen presenting cells (APC) and generate CD4^+^ T cell responses in addition to antibodies. But without access to the cytoplasm of host cells only small amounts of antigen will be available for the induction of cytotoxic CD8^+^ T cells through cross-presentation. This routing of exogenous antigens into the MHC I antigen presentation pathway is only performed by a subset of APC [19]. Partially attenuated vaccines may be able to mimic aspects of the infectious process, such as the initial invasion of host cells, but they do not have the ability to replicate and continue the infection, or infect host cells beyond the nasopharynx (as in the case of the cold-adapted, life-attenuated FluMist^®^ vaccine, which triggers a local and limited infection only [20]). Therefore, even though the same antigens are present in the conventional detergent-split seasonal flu vaccine (e.g., Fluzone^®^) and the live-attenuated influenza vaccine (LAIV), the immune responses they induce are significantly different. Thus, the goal of modern vaccines is to achieve high immunogenicity, but without strong inflammation and, therefore, a good safety profile. An important criterion for the selection of modern adjuvants is their ability to promote the induction of strong CD8^+^ T cell responses. This subset of T cells is able to eliminate cells infected by viruses and other intracellular pathogens, which—once inside cells—are not accessible to antibodies. 

## 3. How Innate Immunity Controls Lymphocyte Responses

**Figure 1:**
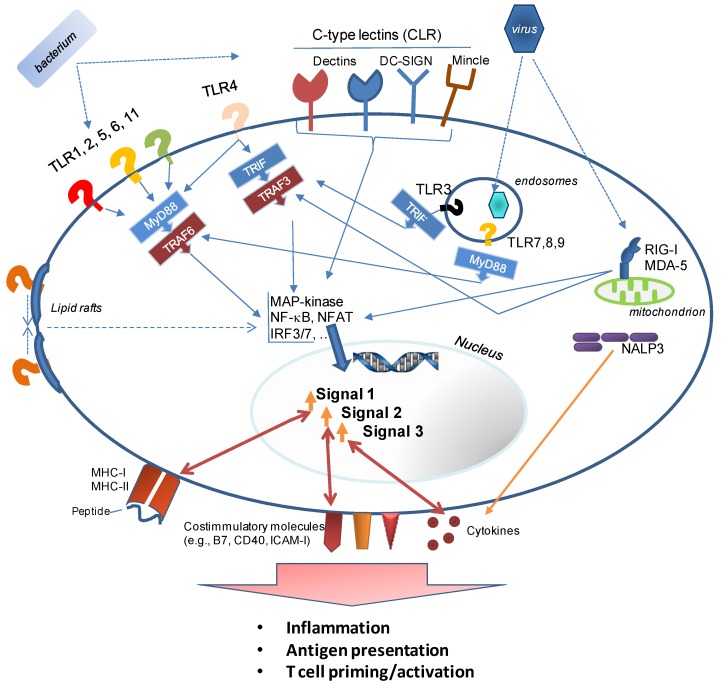
This figure provides a highly simplified overview of Pattern Recognition Receptors (PRR) and the molecular events triggered by the recognition of Pathogen Associated Molecular Patterns (PAMPs) on/inside pathogens. Immune cells as well as many somatic cells express soluble extracellular (not shown), cell surface, and intracellular sentinels for detecting infections. These receptors are specific for distinct classes of pathogen (*i.e.*, bacteria, viruses, fungi, parasites) and are strategically positioned: e.g., receptors that recognize surface components of bacteria such as LPS or LTA are present extracellularly or as soluble molecules (e.g., Mannan-Binding Protein, MBP); receptors for viral RNA are located inside the cell (cytoplasm and phagosome). Binding of PRR to their specific PAMP activates a signaling cascade which relies on common adapter molecules (e.g., MyD88, TRIF), and results in the downstream translation of numerous gene products. Adjuvants emulate these interactions between pathogen and immune system.

For the last couple of decades the trend in vaccinology has been towards simple and more defined vaccines, which are based on select pathogen-derived antigens. Compared to vaccines, which are based on attenuated pathogens, those that use a limited number of pathogen-derived antigens tend to be characterized by a more favorable safety profile and a more straight-forward manufacturing process. They are also based on an improved understanding of immune responses against the pathogens and the knowledge that immune responses against some antigens are more desirable than responses against other antigens such as those associated with immune escape or other undesirable features including cross-reactivity with host antigens. However, using “clean” recombinant antigens removes PAMPs. These conserved, pathogen-derived molecules are recognized by germ-line encoded, evolutionarily conserved innate immune receptors, pattern recognition receptors (PRR, reviewed in [21]), whose presence and activity can transform weakly or non-immunogenic antigens into immunogens capable of triggering T and B cell responses (Figure 1). The inadequate immunogenicity of recombinant proteins as well as carbohydrate antigens has been the driving force behind the search for compounds to endow them with the immunostimulatory capabilities of microbial pathogens, and thus adjuvant discovery. Adjuvant research, however, is more than the identification of novel immunostimulatory molecules. It also includes the proper formulation of the vaccine to achieve maximal immunogenicity.

Ideally, adjuvants trigger multiple, synergistic signaling pathways to achieve optimal adjuvanticity without excessive inflammation. For many adjuvants, no cellular receptors have been identified. Some may activate APC by other mechanisms such as the recruitment of innate immune receptors to lipid rafts. In this scenario, membrane-bound PRR are activated in a ligand-independent manner. Pathogens and properly formulated vaccines trigger multiple signals in an antigen-presenting cells, which result in the induction of a lymphocyte response: Signal 1: is an antigen-specific signal to the T cell receptor through MHC molecules on the surface of the APC. Adjuvants upregulate the expression of MHC molecules and affect the processing of antigens which are presented to T cells. Signal 2: is delivered to T cells through costimulatory molecules, which are upregulated by the APC in response to an adjuvant. Costimulatory signals are essential for the full and effective activation of T cells. Signal 3: is provided to lymphocytes through the establishment of a specific chemokine and cytokine microenvironment. It determines the balance of the lymphocyte response (cellular *versus* humoral), controls the homing routes of lymphocytes and, the type of T helper phenotype of CD4^+^ T cells, and thus the efficacy of the immune response. The choice of adjuvants significantly affects the composition of the cytokine/chemokine milieu created by an activated APC. The initial signaling events triggered by adjuvants for which no specific receptors exist are poorly understood. These adjuvants include crystalline substances (e.g., Alum or crystalline Inulin (Advax^TM^ [17]) or emulsion-based adjuvants. Such adjuvants may trigger the re-arrangement of lipid rafts in the membranes of APCs, resulting in clustering of innate immune receptors [22] and the subsequent signaling from these receptors, but in the absence of a ligand-binding event (as shown for Alum [23]).

### 3.1. Formulation, Formulation, Formulation

Properly formulating adjuvant-antigen combinations in a controlled and reproducible manner is of utmost importance to achieve full and consistent potency as well as long-term stability of a vaccine [6], making “formulation science an unappreciated and often overlooked aspect in the field of vaccinology” [24]. While some adjuvants can be added to an antigen without requiring any specific procedures (such as the ISCOMATRIX™ adjuvant or replication-deficient particles [25,26]), others such as soluble PRR ligands will not induce optimal adaptive immune responses when simply mixed with the antigen [27]. One of the most commonly used adjuvants for both animal studies and human vaccines are aluminum salts, first described by Alexander Glenny in 1926 in the form of “potash alum” [28]. However, the discordance in formulation protocols used by different laboratories to adsorb antigens to alum (or the complete lack of formulation by some), or the use of alum from different manufacturers are a major reason for why immunogenicity data obtained with the “same” vaccine (alum-antigen combination) differ between studies. Different types of alum adjuvants have significantly different adsorption capacity [29]. This would not only affect the amount of antigen being delivered, the release kinetics of the antigen, and thus the quality and intensity of the adaptive immune response, but also dramatically alter the adjuvanticity of co-delivered TLR agonists such as immunostimulatory CpG oligonucleotides [30]. Surprisingly few studies have been published in which different alum formulations are compared side-by-side [31], but the differences in the formulation of alum-adjuvanted vaccines contribute to the inability of alum to serve as a reliable reference or standard adjuvant. The process of “formulation” includes a number of procedures and some selected aspects are discussed below:

(1) The proper attachment of antigens to carriers (nano- or microcarriers such as PLGA beads or aluminum-based crystalline adjuvants); their incorporation into liposomes, virosomes or bacterial ghosts (empty bacterial shells which display bacterial PAMPs [32]); or conjugation to macromolecules (protein, lipid, PEG [33]). These procedures assure co-delivery (mostly to APCs) or protection from extracellular degradation and extended availability (depot);
(2) The correct “assembly” of adjuvant components which, individually, have either no adjuvant activity (e.g., vegetable oil and detergents in the case of nanoemulsions [34], or squalene and detergents in the case of MF59™/AddaVax™ [35,36]), or are very reactogenic (as in the case of hemolytic saponins such as QS21 [37] or Quil A, which are detoxified by their association with cholesterol and phospholipid, forming the adjuvant ISCOMATRIX [38]);
(3) The correct particle size of carriers such as cochleates, which consist of multiple layers of lipid membranes [39], or PLGA microspheres [40]. Much, however, remains to be learned about the optimal size of a vaccine carrier/adjuvant. It depends on parameters such as the route of immunization since different populations of APCs are targeted when the vaccine is injected into different tissues (reviewed in [41]). Not only the particle size, but also the material properties of the carrier appear to affect their immunogenicity [42], making it difficult to establish rules that apply to all particulate vaccine formulations;
(4) Appropriate buffer species (“salting”) with a particular pH and ionic strength [24], since even minor changes in the type of salt used to formulate a vaccine may significantly influence adjuvanticity [43]. The selection of buffers may also impact the stability of the vaccine formulation and, therefore, the immunogenicity of the vaccine;
(5) The spatial arrangement of antigens and/or PAMPs on a particle [44,45] determines how these molecules interact with—and how they cross-link—receptors on APCs such as dendritic cells, macrophages or B cells. The spacing of molecules on a carrier can be precisely controlled for example by using novel programmable DNA nanostructures. On such scaffolds, the impact of incremental changes in molecular distances between molecules (epitope density) can be studied, an approach not adequately used yet to determine the optimal spacing of antigens or PAMPs on a particulate vaccine formulation [46]; and
(6) The ratio of antigen and adjuvant: in most studies, fixed ratios between the two components of the vaccine, adjuvant and antigen, are being used. For each antigen, however, a different ratio may be optimal;
(7) Attenuation of the pathogen: when using killed or partially attenuated pathogens as vaccines, the impact of the attenuation procedure on endogenous adjuvants is rarely discussed or considered. In the case of respiratory syncytial virus (RSV), formalin-based inactivation of the virus unexpectedly created a vaccine that enhanced, rather than prevented disease in RSV-naïve children. The proposed mechanism was a drastically reduced adjuvant effect of viral TLR-ligands, which had been degraded by formalin (reviewed in [47]). The subsequently poor TLR ligation resulted in suboptimal immune responses characterized by low-avidity, non-neutralizing antibodies and T cell-mediated immunopathology [48]. This severe defect of this attenuated RSV vaccine can be overcome by formulating the vaccine with exogenous TLR ligands [49]. It might also be possible to avoid it by employing a milder attenuation protocol, such as the use of hydrogen peroxide instead of formalin. Hydrogen peroxide only causes minimal damage to antigenic structures and thus better preserves the immunogenicity of pathogen-derived antigens [50].

Two examples illustrate how adjuvants can be modified to preserve their adjuvanticity while drastically reducing their reactogenicity:

**Example 1** is the detoxification of LPS, resulting in Monophosphoryl lipid A (MPL^®^) [51] (or its commercially available equivalent, MPLA), a TLR4 agonist, which is safe for use in humans and a component of AS04™. The latter is used in the human Papilloma Virus vaccine Cervarix^®^ (the first FDA-approved vaccine with an adjuvant other than alum). The lower toxicity is a result of much weaker signaling through the MyD88 signaling pathway of TLR4. This signaling cascade activates transcription factors (predominantly NF-κB) associated with inflammatory gene products. Signaling through TLR4’s second signaling cascade, the TRAM/TRIF (Toll IL-1 receptor domain-containing adaptor-inducing IFNβ) pathway is preserved after binding of MPL [52]. TRIF-signaling is associated with a Type I IFN response which is required for the induction of a strong adaptive immune response. However, it should be noted that while the induction of Type I IFN is frequently cited as an indicator of adjuvanticity, at least two adjuvants—the clinically used oil-in-water emulsion MF59 and the TLR-agonist Pam3CSK4—are poor inducers of IFN-related innate immune pathways [53].
  A novel, fully synthetic, mimetic of Lipid A (aminoalkyl glucosaminide4-phosphate (AGP) [54]) has been developed based on the insights into TLR4 signaling pathways gained with MPL. AGP combine the advantages of a highly defined, pure synthetic molecule with the safety of an adjuvant that not only binds to a defined innate immune receptor (TLR4), but also preferentially triggers a beneficial innate immune response profile (TRIF-signaling and Type I IFN production) while avoiding a signaling cascade which results in excessive inflammation (MyD88 signaling). While this development is very encouraging, it is important to evaluate the immune responses induced by the modified and “safer” adjuvants to determine whether any benefits of the “stronger” adjuvants had been lost. In the case of MPL, it appears that both LPS and its derivative promote strong CD4^+^ T cell expansion, but long-term retention of these cells was only supported efficiently by LPS [55].
**Example 2** is the “decoration” of antigens with small-molecule adjuvants. This is a formulation approach which drastically reduces the amount of adjuvant being delivered and thus curbs the inflammatory response while selectively and efficiently triggering the activation of PRR on those APCs which encounter the antigen. This approach was used for a synthetic TLR7 agonist, imidazoquinoline [56], and resulted in the induction of a robust, high-affinity antibody response. The direct conjugation of antigen to another TLR-agonist also supported the induction of CD8^+^ T cell responses (further discussed below), likely by enhancing cross-presentation [57]. While this approach is only limited by the creativity and skill of the medicinal chemist, the product has to be evaluated very carefully to ensure that immune responses to the “decorated” antigen are not negatively affected by PAMPs attached to crucial T or B cell epitopes. Masking of a crucial epitope for broadly neutralizing antibodies was indeed observed after a gp120 HIV vaccine was decorated with TLR-agonists [58]. This problem may be avoided by conjugating both adjuvant and antigen to a carrier (such as nanolipoprotein particles [59]).

### 3.2. Adaptive Correlates of Adjuvanticity—What Is the Best Readout?

When judging the effectiveness of a compound to act as a vaccine adjuvant, most researchers determine antigen-specific antibody titers. This readout may not be meaningful in all cases, since vaccine efficacy does not necessarily correlate with bulk antibody titers, but may depend on the fine specificity and quality of the antibodies or cellular immune responses. Protection against many diseases may require a strong CD8^+^ T cell-component and/or a very specific antibody isotype profile. In addition, improved antibody avidity and epitope spreading generally improve vaccine efficacy by providing more robust protection as well as the potential for cross-protection. The latter is a particularly desirable feature of vaccines against highly variable pathogens such as HIV or influenza. The oil-in-water adjuvant MF59 has been shown to trigger both epitope spreading and an increase in antibody avidity when added to an H5N1 influenza vaccine [60]. Similarly, an imidazoquinoline dendrimer-based adjuvant promoted epitope spreading and higher affinity antibody responses when compared to a vaccine containing the same amount of the monomeric TLR7/8-agonist (imidazoquinoline). Even though the dendrimer had lost the ability of its monomeric components to bind to TLR8, it was still able to signal through TLR7 [61]. This maintained the ability of the molecule to provide strong adjuvanticity, while lacking the undesirable inflammatory profile of a TLR8 agonist which has been linked to the induction of autoimmunity [62]. 

Such observations underscore (1) the ability of adjuvants, when properly selected and formulated, to enhance adaptive immunity without necessarily increasing reactogenicity; and (2) the need for more comprehensive evaluations of immune responses induced by vaccines with novel adjuvants. Ideally, adjuvants are selected based on their ability to enhance responses that are associated with protective immunity against a particular pathogen. Unfortunately, these correlates of protection are unknown for most pathogens. Routinely comparing several adjuvants side-by-side when developing vaccines not only reduces the risk of vaccine failure due to the choice of a “wrong” adjuvant, but comparing immune profiles induced by various formulations with the same antigen may accelerate the identification of elusive immune correlates.

## 4. What Does an Adjuvant Do and How Does It Affect Adaptive Immune Responses?

Most immune potentiators are ligands of PRRs and many of them represent molecules that are uniquely associated with microorganisms (PAMPs). Host-derived Damage Associated Molecular Patterns (DAMPs), which can also activate innate immune responses, have only recently been explored as vaccine adjuvants, such as the High Mobility Group Box 1 Protein [63]. Initially, PAMPs were isolated from pathogens and used in their original form. Excessive inflammatory responses prompted the development of the next generation of PRR-agonists in the form of modified versions of naturally-occurring PAMPs (e.g., conversion of highly reactogenic LPS into the “low-toxicity” MPL discussed above). The current trend in the adjuvant field is towards synthetic PRR agonists. These are (1) compounds which either mimic the natural ligand and, by being synthetic, circumvent the various problems associated with the isolation, purification, and modification (such as heterogeneity, batch comparability, cost) of the naturally occurring PAMP; or (2) compounds with little or no structural similarity to the natural ligand, which were identified by screening large libraries of chemical compound [64,65,66]. In all instances, binding to the innate immune receptor triggers a molecular signal, resulting in the activation of the targeted cell (in most cases a leukocyte although non-hematopoietic cells may also express PRRs and respond to adjuvants [67]), and an inflammatory microenvironment. While antigen recognition by lymphocytes in the absence of inflammatory signals results in acquired non-responsiveness (immune tolerance), an inflammatory environment promotes the induction of an adaptive (lymphocyte) response. Although this basic model of immune activation is undisputed, understanding which inflammatory signals and factors are actively involved and responsible for adjuvanticity is rudimentary at best. 

### 4.1. Correlates of Adjuvanticity—Soluble Factors?

The mechanism of action of an adjuvant is often described in terms of the chemokines and cytokines it induces, at the injection site, within the draining lymphoid tissue, or systemically. Chemokines, which promote immune cell migration, are undeniably crucial for adjuvanticity and have therefore been used themselves as vaccine adjuvants (reviewed in [68]), but the measurement of individual or even several of these factors has limited value when explaining how a compound provides an adjuvant effect for a co-delivered antigen. Adjuvants trigger a chemokine-driven immune amplification loop (reviewed in [69]) and the importance and role of individual factors is not easy to decipher. Cytokines produced by activated lymphocytes are also frequently used to gauge the immunogenicity of a vaccine. In animal studies, IFNγ is commonly measure and the BCG vaccine against tuberculosis has been described as the only licensed vaccine to mediate its protective efficacy through this cytokine. In this case, IFNγ is produced by vaccine-induced CD4^+^ T cells, activating macrophages which in turn kill the bacteria. However, this model was recently challenged, calling into question the value of IFNγ as a marker of vaccine-induced immune protection [70]. A panel of cytokines is produced in response to stimulation of immune cells by adjuvants and efforts are underway to establish thresholds of “safe adjuvanticity” based on levels of factors such as IL-β, IL-6, TNF-α, and IL-8 [71]. What is not clear yet, however, is which factor contributes to immunogenicity *versus* reactogenicity. Crucial parameters are likely not just the concentrations of the individual factors, but the interplay between multiple factors. Studies such as the comparison of the innate immune responses induced by the highly reactogenic LPS and its low-toxicity derivative MPL (see below) provide valuable insights into the importance of individual soluble factors for adjuvanticity. Such insights may not be obtained when comparing the response profiles of adjuvants from different classes (such as the comparison of alum with a TLR agonist). 

### 4.2. Correlates of Adjuvanticity—Leukocyte Recruitment?

Another frequently reported mechanism of adjuvants is the attraction of a particular cellular infiltrate to the site of the injection or the draining lymphoid tissue [26,72,73]. Mobilization, activation, and attraction of leukocytes are initiated and controlled by chemokines and unquestionably correlate with the immunogenicity and efficacy of a vaccine. However, the cellular infiltrate at the injection site of a vaccine may contribute to adjuvanticity as well as reactogenicity. The key question is how many cells of a particular cell type are required for optimal adjuvanticity. Neutrophils are among the very first cells infiltrating an injection site and in side-by-side comparisons more potent adjuvants appear to attract more neutrophils. Unexpectedly, however, ablation of this cell type did not affect the immunogenicity of MF59 [72], calling into question their relevance for adjuvanticity. Neutrophils have even been reported to negatively affect aspects of adaptive immune responses following vaccination with antigens plus alum or Freund’s complete adjuvant [74]. In contrast, the depletion of CD11c^+^ monocytes and dendritic cells (DCs) during immunization with an alum-adjuvanted vaccine abrogated both antibody and cellular responses [75], identifying these cell populations as essential for immunogenicity at least in the context of this particular vaccine formulation. 

### 4.3. Correlates of Adjuvanticity—The T Cell Perspective

The effects that adjuvants have on the innate immune system are broad and diverse and how these affect T cells is equally complex. Different adjuvants result in T cell responses with different intensity (size of T cell pool, selection of T cells with particular TCR affinity), and character (CD4^+^ or CD8^+^ T cell, CD4^+^ T helper subtypes Th1, Th2, Th17, Tfh (T follicular helper cells)), depending on the following: (1) whether antigen and adjuvant are co-delivered to the same phagosome. This can be controlled through the formulation of the vaccine. Co-delivery of antigen and adjuvant has a direct impact on antigen presentation and thus TCR affinity and repertoire (reviewed in [76]); (2) the panel of chemokines and cytokines released in response to a particular adjuvant, which changes the activation status of surrounding cells including APC and thus impacts the efficiency of antigen processing and loading of MHC molecules for presentation to T cells; (3) adjuvants and the formulation of the vaccine determine the persistence of antigen and, therefore, duration of antigen-presentation which in turn directly affects T cell activation; (4) adjuvants may recruit other APC such as B cells which, depending on their processing ability and costimulatory signals (specifically signal 3, the cytokine response), determine the type of T cells response induced; and (5) changes in the antigen such as chemical modification to form aggregates or generate fusion partners with APC-targeting moieties impact the fine specificity of the T cell response by changing the hierarchy of immune-dominance, revealing cryptic epitopes and in some cases also generating neo-epitopes (which may be an undesirable outcome).

### 4.4. Polarization of T Cell Responses towards Cellular or Humoral Responses

Immune correlates of protection are still either unknown or poorly characterized for most pathogens, making vaccine development a largely empirical process. Protection against many pathogens, however, depends on the induction of both, humoral and cellular responses. CD4^+^ T cells are required for the induction of efficacious adaptive immunity although the protective CD4^+^ T cell phenotype and cytokine profile remains to be identified for many pathogens. In the case of responses requiring neutralizing antibodies, CD4^+^ T cells provide T cell help to B cells for full activation and to endow them with the ability to further differentiate into either antibody producing cells (plasma cells) or memory B cells. T cell help is also required for efficient affinity maturation and isotype switching of antibodies. Relatively few adjuvant studies include the analysis of antibody avidity as a measure of adjuvant efficacy, but this additional analysis adds valuable information which helps with the selection of adjuvants and vaccine formulations [77]. Antigen-specific antibody titers alone are an insufficient correlate of vaccine efficacy. The type of helper T cell response induced determines the isotype profile of the antigen-specific antibodies. Information about which isotype profile is associated with protection against a particular pathogen is still not available for many diseases but this information may guide the selection of adjuvants based on the ability of the immunostimulatory compound to preferentially induce a T helper subtype. CD4^+^ T cell help is generally also required for the induction of antigen-specific CD8^+^ T cells, as well as their full activation and differentiation into effector and/or memory cells. Most adjuvant studies focus on the short-term enhancement of T cell responses and often fail to ask how well this T cell response is maintained. The above-mentioned comparison of LPS and its detoxified derivative MPL indicated that only long-term retention, but not the short-term expansion of CD4^+^ T cell was negatively affected when the pro-inflammatory MyD88 signaling pathway was triggered less by MPL than by LPS [55]. Well-established adjuvants such as the mucosally-delivered Cholera toxin are clearly able to promote long-term immunological memory [78]. However, considerations such as the high costs of long-term immunogenicity studies appear to stand in the way of the answer to this important question which can determine whether or not a vaccine will ultimately be successful in humans or not.

### 4.5. Alum—The Stumbling Block for Better Vaccines?

The Th2-bias of immune responses induced by alum-adjuvanted vaccines is one of the most cited observations in the vaccine literature and is a feature that may limit the development of vaccines against a variety of pathogens. Nevertheless, the use of alum as the adjuvant of choice is likely to continue due to the favorable safety record of aluminum-based adjuvants. In addition, novel compounds require a lengthy approval process [79]. However, there is a surprising scarcity of reports describing a vaccine-mediated induction of robust CD8^+^ T cells responses with adjuvanted vaccines in humans. In a setting where CD8^+^ (Tc1) effector cells are crucial for protection–such as therapeutic cancer vaccines–one approach to overcome this problem has been the use of peptide vaccines with “strong” adjuvants to force the induction of a T cell population (particularly CD8^+^ T cells) with defined specificity. This approach had only limited success and only occasional objective clinical responses have been reported [80]. Various factors contribute to the poor immunogenicity and efficacy of peptide vaccines, including the instability of peptide vaccines in the presence of serum [81] and the fact that an epitope-based vaccine is subject to HLA-restriction. Moreover, peptide vaccines are at risk of escape mutants where a single mutation within the tumor antigen or down regulation of the expression results in the inability of the peptide specific T cells to recognize and eliminate the tumor cells. Finally, a peptide vaccine representing only the CD8^+^ T cell epitope suffers from lack of T cell help resulting in poor immunological memory. Therefore, there is an unmet need for safe vaccines capable of inducing strong CD8^+^ T cells responses and this need will have to be met by novel adjuvants. Alum may, after all, be useful as a vaccine adjuvant of the future when used in combination with other adjuvants such as MPL (further discussed below). 

### 4.6. Wanted—Adjuvants That Induce CD8^+^ T Cells

Currently, the development of formulations capable of inducing robust cellular responses in humans is one of the great challenges for vaccinologists. The only licensed vaccines capable of generating long-lived cellular immunity are live attenuated pathogens and the challenge is to develop formulations capable of replicating key signaling events and cellular processes triggered by those successful vaccines, such as the yellow fever vaccine [82]. DNA-prime/viral-vector-boost strategies have been used to induce CD8^+^ T cell responses in humans with both types of vaccines capable of routing proteins through the MHC I antigen processing pathway. Boosting with a viral vector overcomes the often-insufficient immunogenicity of DNA plasmids, and priming with a DNA vaccine reduces the risk of inducing neutralizing antibodies against the viral vector. However, the more complicated heterologous immunization regimen makes this an unattractive approach for many vaccines. 

Choosing an adjuvant for a vaccine meant to induce strong T cell responses does not necessarily mean selecting from a list of “proven” T cell adjuvants. The ability of a vaccine to (a) induce a T cell response and (b) a specific type of T cell response (CD4^+^/CD8^+^; Th1/Th2/Th17) depends on a variety of factors such as the nature of the antigen, the vaccine platform (recombinant protein *vs.* gene-based vaccines such as DNA or recombinant virus), the immunization regimen (homologous *vs.* heterologous prime-boost), the route of administration, the immunization interval, and the frequently overlooked importance of formulation. For example, MPL, a strong adjuvant for both animal models and humans, potently enhances antibody responses when used as a soluble molecule, but strongly enhances T cell responses when formulated in oil as in the liposome-based AS01 or the oil-in-water emulsion AS02, the latter being a good inducer of cytotoxic T cells (reviewed in [83]). The following are a few select adjuvants reported to induce T cell responses:

- TLR9 is an intracellular sensor of dsDNA characterized by a central, non-methylated CpG and flanking sequence motifs. Synthetic CpG oligonucleotides (ODN) have been reported to induce strong cytotoxic T cell and Th1 responses [84], and also support a robust antibody response. Not only are CpG motifs species-specific (complicating their translation from small-animal models to the clinic), but different motifs target different populations of APCs [85] and trigger different response profiles [86].
- TLR5 is a membrane-based receptor for bacterial flagellin. Vaccine constructs consisting of antigen-flagellin fusions induce balanced Th1 and Th2 responses [87]. Flagellin has already been tested as an adjuvant for a novel influenza vaccine in humans [88] and in addition to its exploration as a vaccine adjuvant for a variety of infectious disease vaccines in animal models, it has been able to enhance papilloma virus-specific CD8^+^ T cell responses in a therapeutic cancer vaccine model [89] or CD8^+^ T cells associated with protective immunity in a malaria model [90].
- TLR7 and TLR8 are intracellular sensors of single stranded RNA and ligands for these PRR have been used in the clinic for topical treatment of various types of skin cancer [91]. Numerous agonists have been developed (reviewed in [92]), such as the TLR7-selective and Th1/Th17 polarizing guanosine-analog Loxoribine [93]. Signals through TLR7 have been found to promote cross-presentation by dendritic cells (DC), thus enhancing the induction of CD8^+^ T cells [94]. Testing of TLR7 or TLR8 agonists is complicated by the fact that the cellular distribution of TLR7 is significantly different between mice and humans, and the ligand specificity of TLR8 is drastically different between the two species (leading to the initial conclusion that mouse TLR8 was not functional).
- Numerous reports have documented the usefulness of the TLR3 agonist polyriboinosinic acid-polyribocytidylic acid (poly(I:C)) to induce cellular immune responses. The synthetic RNA molecule allows the induction of CD8^+^ T cells against soluble proteins in mice [95,96] and has been added to DC-based vaccines [97] or peptide-vaccines [98] for cancer immunotherapy. It induces strong CD8^+^ T cell responses against the co-delivered HIV Gag protein and this response was shown to be further improved through the addition of ISCOMs. Combining the two types of adjuvant provided a synergistic adjuvant effect [99].
- Among adjuvants with unknown receptor specificity or a defined mechanism-of-action, ISCOM-based formulations have been used to induce antibodies as well as CD8^+^ T cell responses, either in the form of ISCOMATRIX, which is simply added to the antigen or as ISCOM-based vaccines. In the latter, the antigen is encapsulated within nanocages consisting of saponin, cholesterol, and phospholipid. This type of adjuvant is a component of two veterinary vaccines and has also proven to be efficacious in clinical trials (reviewed in [100]). Not surprisingly, strategies to enhance the association of free antigen with ISCOMATRIX (such as increasing the electrostatic interaction between protein and the nanocage) result in stronger CD4^+^ and CD8^+^ T cell responses, as shown, for example, with an HCV vaccine in primates [101].

### 4.7. Wanted—Adjuvants that Induce Better CD8^+^ T Cells

While alum by itself is a famously poor inducer of CD8^+^ T cell response, it continues to be used in modern adjuvant formulations as a co-adjuvant or carrier of more potent inducers of cellular immune responses. Alum can (1) slow the diffusion of small-molecule PRR-agonists thus reducing their systemic effects; and (2)—based on well-understood pharmacokinetics—deliver both PRR-agonist and antigen to draining lymph nodes. How it provides a co-adjuvant effect by synergizing with the co-delivered adjuvant is still not understood, which is not surprising since the mechanism of action of alum alone is still controversial (discussed below). Several adjuvants have been combined and formulated to induce robust T cell responses; for example:

- MPL, the derivative of the bacterial TLR4 agonist LPS, has been used alone as well as in combination with other adjuvants such as QS21 in liposomes (AS01) or an emulsion (AS02), alum (AS04), or CpG and QS21 in an emulsion (AS15) with the goal of promoting T cell responses (MPL has been used in millions of doses of vaccines (licensed products as well as experimental vaccines). These vaccines include Fendrix^®^ (HBV), Cervarix^®^ (HPV), RTS,S (malaria; final stages of licensure), Pollinex Quattro^®^ (allergy)). A related LPS derivative, Glucopyranosyl Lipid Adjuvant (GLA) [102], formulated in a stable emulsion (SE), which by itself has adjuvant properties, has been used as an adjuvant for experimental, clinical vaccines against Leishmania, Influenza, TB, and malaria (reviewed in [7]). TLR4 promotes B cell maturation [103], changes the trafficking of B cells into the germinal centers of lymphatic organs [104], and likely regulates affinity maturation [105]. In the context of T cell responses, it should be noted that TLR4-signalling mediates the trapping of activated CD8^+^ T cells in the liver [106]. Depending on the targeted disease, this could be advantageous when effector T cells accumulate at the site where the pathogen resides (e.g., in the case of Hepatitis or malaria). However, this is based on the assumption that T cell function is retained in the liver which has been described as a lymphoid organ with suppressive rather than stimulatory characteristics (reviewed in [107]).
- Combining CpG ODN with alum improves both antibody and T cell responses [108].
- Phytol is a branched aliphatic alcohol and a constituent of chlorophyll. Synthetic, modified phytols are potent immunostimulatory molecules and can be used to drive Th2 or Th1 responses, depending on the specific chemical modifications [109,110]. These compounds promote humoral immune responses as well as the induction of potent CD8^+^ CTL responses [111]. Although the mechanism of phytol-based adjuvants is still unknown, and specific cellular receptors which mediate their function(s) remain to be identified, these compounds are a reminder that natural compound libraries likely contain many novel adjuvant candidates waiting to be discovered.

### 4.8. What Does It Take to Activate CD8^+^ T Cells?

Bouvier *et al.* reported that prolonging antigen release (“depot effect”) greatly enhances cross-priming and induction of CD8^+^ T cells [112], indicating the need to formulate the antigen not only with a strong adjuvant but also a carrier that accomplishes the delayed release. As discussed below, alum does not provide a substantial depot effect though this is one of its most cited mechanisms of action. Not surprisingly, alum is a poor inducer of CD8^+^ T cells, but, surprisingly, another aluminum-based nanoparticle, *α*-Al_2_O_3_ efficiently enhanced CD8^+^ T cell induction [113]. Polymerosomes, stable vesicles consisting of block copolymers, which release antigens as well as co-deliver adjuvants in a highly predictable manner, also promote cross-presentation [114]. Even in the absence of additional ligands of innate immune receptors, antigens bound to particles have been used to induce both strong CD8^+^ T cell and humoral responses [115]. Providing a depot effect certainly is not the only path to strong CD8^+^ T cell induction. ISCOMATRIX-adjuvanted vaccines are rapidly removed from the injection site [25] and thus provide virtually no depot effect, but induce strong CD8^+^ T cell responses through promoting cross-presentation [116,117]. Since modern (recombinant or subunit) vaccines represent non-infectious material, the antigens are mostly routed through the MHC-II, but not the MHC-I, antigen processing pathway. Thus, such vaccines have to rely on a secondary pathway for cross-presentation of exogenous antigens [19]. Any modification of a vaccine/vaccination procedure that promotes cross-presentation should be considered when trying to enhance induction of CD8^+^ T cells (reviewed in [112]). These include strong T cell help leading to the licensing of DCs, which could be accomplished with appropriate adjuvants. Various adjuvants have been reported to promote cross-presentation, although how they compare to each other is difficult to judge when they are tested in different experimental systems. They include the direct conjugation of antigen to TLR-agonists, which results in efficient CD8^+^ T cell induction [57]. Not surprisingly, when considering the importance of CD8^+^ T cell responses against viral infections, sensors of viral PAMPs have been reported to assist in the induction of this lymphocyte population. TLR7 stimulation by polyuridylic acid (polyU), a synthetic ssRNA analog, stimulates cross-presentation [94]. Another study combined aggregation of antigen and signaling through TLR7/8 to induce strong T cell responses [118]. dsRNA, mimics viral genomic nucleic acid and binds to TLR3 (and other RNA-sensors such as RLRs, depending on the length of the RNA ligand). It supports strong cross-presentation and adjuvanticity [119], but results obtained with another synthetic TLR3, poly(I:C), are less clear. TLR3 and RLR ligands have been reported to induce IL12, type I IFN, and promote cross-presentation, all of which explain their ability to induce CD8^+^ T cell responses (reviewed in [120,121]). Poly(I:C) has already been used in clinical trials and some reports show good induction of T cells, while others have noted that pre-treatment of the vaccine recipient with poly(I:C) may actually inhibit cross-presentation [122]. Also, when poly(I:C) was added to an Ad26-vector-based vaccine, CD8^+^ T cell responses were suppressed, not enhanced [123]. In the same study, the addition of a TLR4 agonist enhanced CD8^+^ T cell responses. A similar result was obtained when an adenovirus-based tumor vaccine was further adjuvanted with a TLR9 agonist, resulting in reduced CD8^+^ T cell induction [124]. Surprisingly, in this model, anti-tumor responses were still stronger, indicating mechanisms other than CD8^+^ T cell-mediated immunity for protection. A potential reason for the suppression of CD8^+^ T cell responses, induced by antigens encoded by viral vectors may be the suppression of antigen expression by antiviral signaling pathways, a mechanism that is helpful when controlling a viral infection, but which would affect any vaccine that requires the *de novo* expression of antigen, such as recombinant viruses or DNA vaccines. Indeed, certain adjuvants have been noted to reduce antigen expression at the injection site after DNA vaccination which did not, however, reduce vaccine immunogenicity. This is likely due to the presence of the adjuvant which efficiently increased the immunogenicity of the small amounts of antigen that were produced (dose sparing effect of adjuvants). 

### 4.9. Inducing the Strongest CD8^+^ T Cell Response—A Good Idea?

While the main objective of a vaccine is to induce protective immunity against infection (or therapeutic efficacy in the case of established disease, such as cancer), the efficacy of adjuvants is most frequently judged based on their ability to induce potent adaptive immune responses. Excessive stimulation of lymphocytes, however, may not be desirable. A study using a peptide vaccine formulated in incomplete Freund’s adjuvant stimulated, as expected, a potent CD8^+^ T cell response. Interestingly, very few of the T cells reached the tumor they were specific for and many died at the injection site [125]. The vaccine-induced retention and dysfunction of antigen-specific CD8^+^ T cells was also observed in patients immunized with a melanoma vaccine formulated with the same adjuvant [126]. Overstimulation of T cells has also been reported to result in activation-induced death, but this phenomenon has not been explored extensively in the context of vaccine adjuvants. Preventing the premature death of effector lymphocytes and extending their window of activation can significantly enhance the efficacy of vaccines. Novel types of adjuvants which interfere with regulatory (“shut-down”) pathways, such as small interfering RNA (siRNA) that inhibits Suppressor of Cytokine Signaling (SOCS) 1, are currently being explored [127].

## 5. The Confusing (Molecular) Mechanism of Action of Well-Known Adjuvants

### 5.1. What Is an “Adjuvant Effect” on a Molecular/Cellular Level?

Various types of cells, most prominently APCs such as DCs, respond to many adjuvants and the upregulation of activation markers or secretion of cytokines from leukocytes after exposure to such substances can easily be observed and quantified. These parameters are frequently used to study the effects of vaccine adjuvants. The following two issues need to be considered: First, in many cases, “adjuvant” activity is initially identified by *in vitro* screening based on reporter cell lines expressing individual or multiple innate immune receptors. While such approaches are the only realistic way to screen large numbers of compounds for adjuvant activity, the *in vitro* response of individual cells does necessarily correlate with *in vivo* adjuvanticity. Improved *in vitro* screening systems, which better mimic the complexity of immune responses, are sorely needed and mixed cell cultures of APC and stromal cells are starting to address this issue. Second, isolated cellular responses are only a downstream aspect of the mechanism of action of adjuvants. The underlying cellular signals and—in many cases—the cellular receptors for adjuvants which initiate the response, are unknown or only poorly understood. This is particularly true for adjuvants that fall into the category of “delivery vehicles”. Emulsions (such as Freund’s adjuvant or MF59) do not bind to dedicated cellular receptors. In the case of insoluble aluminum-based adjuvants (“Alum”), such receptors would not be predicted to even exist [23]. Nevertheless, the oil-in-water emulsion MF59 mediates its adjuvanticity through MyD88 [128]. Although MyD88 is the adaptor molecule for multiple TLRs, MF59’s adjuvanticity does not depend on TLR. The same is true for complete Freund’s adjuvant, which induces Th17-differentiation and IL-17-secretion in a MyD88- and IL-1β-dependent manner [129], although in the case of this more complex adjuvant, mycobacterial peptidoglycan was the major factor responsible for inflammasome activation and IL-1 production. Even the nanocages of the ISCOMATRIX adjuvant rely on MyD88 for their effectiveness [117], although no TLRs appear to be involved. 

While it may seem that mainly those adjuvants which have no known cellular receptors pose a challenge when trying to decipher their molecular and cellular mechanisms of action, even ligands for PAMPs, with their more-or-less well-described signaling pathways are not necessarily “predictable”. TLR4 is the best characterized TLR and it is unique among the TLRs since it signals through both, the MyD88 and the TRIF pathway, with both contributing to adjuvanticity [130]. However, minor changes in the structure of the ligand can determine which pathway is favored [54], the pro-inflammatory MyD88 or the more beneficial TRIF pathway [131] as discussed above. Furthermore, it was only recently recognized that LPS, the prototypic TLR4 agonist, additionally signals through a TLR4 independent, Caspase-11-dependent mechanism [132], suggesting that other, unknown signaling pathways may also be involved in the mechanism of ligands for supposedly well-defined innate immune receptors.

### 5.2. Alum—The Never Ending Story

Although aluminum salts have been used as vaccine adjuvants in humans for almost a century—and remain the adjuvant of choice for novel vaccines because of their strong safety profile and history—their mechanism of action continues to puzzle researchers [133]. In part, the conflicting observations made by various investigators are due to the use of different formulations of alum, the antigen used, the animal model, and the immunological readout of the vaccine-induced response [31], and thus hampers comparative adjuvant research in general. The concept that alum works by providing a depot effect, slowly releasing the adsorbed vaccine antigen from the injection site, has been the most persistent dogma, although it has been debunked recently (reviewed in [134]). Experiments involving the removal of the injection site clearly show that the inoculum does not have to persist in the periphery in order for alum to work its magic [135]. 

Various studies describe the uptake of alum particles into the phagolysosome of antigen processing cells, resulting in the delivery of adsorbed antigen into the MHC-II processing pathway. However, when comparing different types of APCs, only macrophages—but not DCs—appeared to ingest alum [23]. In the case of DCs, the adjuvant particles strongly interacted with the cell membrane, resulting in antigen “transfer” to the APC, rearrangement of lipid rafts in the DC’s membrane and activation of cellular receptors without the alum ever entering the cells. This unexpected finding highlights a mechanism that might also contribute to the efficacy of various other adjuvants, but has not been thoroughly studied: emulsion-based adjuvants, too, may trigger the re-arrangement of membrane micro-domains such as lipid rafts on leukocytes. Surface-expressed immune receptors display a non-random spatial distribution (reviewed in [136]) and their rearrangement may result in intracellular signals, which could resemble those induced by ligand-mediated receptor ligation. The induction of inflammatory processes through the recruitment of TLR4 and TLR2 to lipid raft-caveolae, followed by their activation in the absence of TLR-ligands, was observed in response of cells to ethanol and provides an unexpected explanation for inflammatory disease associated with alcoholism [22].

### 5.3. Nalp3 Inflammasome—The Missing Link?

In 2008, several groups appeared to have solved the mystery of alum’s adjuvanticity [137,138,139]. The new model involved the uptake of alum into phagolysosomes, followed by destabilization of the vesicles and the release of cathepsin into the cytoplasm. There, a NOD-like receptor (specifically, NLRP3 (Cryopyrin/NALP3/Pypaf1)), which acts as a sensor for a variety of stimuli (including a number of pathogens) assembles with the final NALP3 inflammasome complex and activates Caspase-1. This cysteine protease in turn cleaves pro-IL-1β, completing a cascade of cellular events that culminates in the release of biologically active IL-1β and, thus, an inflammatory response. The model was quite appealing since other crystalline substances such as uric acid or silica, also appear to signal through this pathway, providing a mechanistic explanation for the inflammatory disease associated with the two substances, gout and silicosis of the lung. Not all crystals, though, are sensed through this pathway since aluminum powder or diamond crystals do not appear to activate NALP3. A possible explanation for this observation is provided by a more recent finding that the surface texture of a particle determines its ability to trigger inflammasome activation and, thus, the immunogenicity of particulate material [140]. Rough surfaces are associated not only with stronger inflammatory responses, but also better uptake through phagocytosis and neutrophil recruitment. 

However, the results from studies advocating for a central role of the NALP3 inflammasome in alum’s adjuvanticity were partly contradictory and *in vitro* findings were inconsistent with *in vivo* observations: unlike *in vivo*, alum does not activate DCs *in vitro* and pro-IL-1β is only expressed in response to an alum-independent stimulus, such as the TLR4-stimulation by LPS. Differences in the *in vivo* results of different studies might be explained by the same issues which complicate the comparison of adjuvants in general: (1) the formulation of the vaccine (in this case, the efficiency of the adsorption of antigen to alum or the type of alum used [30]); (2) the antigen dose; (3) the immunization regimen; (4) the immunological readout; or (5) the animal model used (especially, species and strain, but also gender and age. An only recently recognized modulator of immune responses is an organism’s microbiome. In the case of laboratory animals, the microbiome can vary significantly between animal facilities). In regards to the immunological readout, it makes a significant difference whether cellular or humoral responses (in particular the isotype profile of antibody) are measured. Injection into different tissues results in the targeting of different populations of APCs. Finally, the nature and purity of the antigen can drastically affect the adjuvanticity of a vaccine formulation: some proteins are inherently immunogenic, such as the cancer/testis antigen NY-ESO that unexpectedly triggers TLR4 signaling [141]. Recombinant protein preparations are frequently contaminated with endotoxin (LPS), and even small amounts can provide significant adjuvanticity. The effect of the LPS-“co-adjuvant” can be amplified if the adjuvant which was purposely added to the formulation triggers a synergistic signaling pathway [142]. This co-adjuvant effect of an LPS-contaminant at low levels, if properly controlled, may thus even be viewed as a beneficial component of a vaccine [143].

### 5.4. Death by Alum

Uric acid was implicated in the mechanism of alum’s adjuvanticity [144], suggesting that this inflammasome activator was released by cells killed after exposure to alum, thus providing an indirect activation of the inflammasome by alum. Although uric acid may not be essential for the adjuvanticity of alum after all, this study [144] did, however, highlight the importance of adjuvant-induced cell death. The impact that cell death has on immunogenicity has been known for more than a decade (reviewed in [145]), although the overly simplistic model that apoptosis is associated with tolerance while necrosis promotes inflammatory responses and immunity was recently revised. Alternate forms of cell death, such as pro-inflammatory, RIP-kinase-dependent necroptosis (reviewed in [146]) or Caspase-1—dependent pyroptosis (reviewed in [147]) were added to the various mechanisms by which a cell can die. The importance of cell death for vaccine efficacy was previously described for DNA vaccines [148] and enhancers of targeted cell death induced by the DNA vaccine have already been explored as molecular adjuvants (reviewed in [149]). Surprisingly, however, the induction of cell death by a candidate adjuvant is still frequently used as an exclusion criterion when evaluating novel adjuvant candidates. Adjuvant-induced cell death is often considered an undesirable side effect despite the well-characterized link between TLR-activation and cell death (reviewed in [150]) with at least 7 out of 10 human TLR having pro-apoptotic activities. In the case of TLR7, this pro-apoptotic activity is used to treat basal carcinoma, transformed keratinocytes, or melanoma with Imiquimod [147,151]. Several adjuvants have been reported to induce cell death, including nanoemulsions, which consist of “benign” components, namely vegetable oil and surfactants, and which trigger the death of ciliated nasal epithelial cells when delivered intranasally [34]. Another candidate mucosal adjuvant, Polyethylenimine (PEI), a family of organic polycations, was also shown to mediate cell death [152]. While the precise type of cell death induced by nanoemulsions has not been elucidated yet, they do trigger the engulfment of dying cells by DCs and activation of T cell responses. The link between cell death induced by the adjuvant, and antigen uptake by DCs, and the subsequent induction of T cell responses, has also been shown for tomatine adjuvant [153]. The benefit of cell death induced by alum appears to involve the release of host cell DNA [154], a well-described DAMP. When comparing these studies it is important to note that different types of cells, different conditions and different assays to report cell death were used, making a true side-by-side comparison of the ability of different types of adjuvants to induce different types of cell death virtually impossible.

### 5.5. New Players in the Model of Alum’s Mechanism

The questions that remain for alum are (1) how does alum kill cells, and (2) how does the frequently cited activation of the NALP3 inflammasome contribute to the process? The model which proposes a central role for NALP3 in the adjuvanticity of alum is based on activation of the inflammasome following the disruption of lysosomes by alum, thus describing NALP3 as a sensor of lysosomal damage and, therefore, as an endogenous danger signal [155]. Following lysosomal disruption, cathepsins are released into the cytoplasm. Cathepsins not only process key molecules of the apoptotic machinery [156], they also degrade components of the NALP3 inflammasome, thus interfering with the proposed key mechanism of adjuvanticity [157]. However, if the rupture of lysosomes and release of cathepsins is a “switch” which inevitably leads to cell death, why don’t stimulators of this pathway all trigger the exact same result in terms of the type of cell death? While the extensive and complete rupture of lysosomes results in necrotic cell death, partial and selective lysosome permeabilization triggers apoptotic cell death [158]. The two forms of cell death have different immunological consequences. A side-by-side comparison of known NALP3-inducers (ATP and nigericin) and lysosome disrupting agents (alum and Leu-Leu-OMe (LLOMe)) revealed the drastically different pathways induced by the two types of compounds. This finding further undermines the popular hypothesis that alum mediates its adjuvanticity through the NALP3-inflammasome: the inflammasome inducers triggered the expected IL-1 release from stimulated cells, together with caspase-1-dependent pyroptosis in the absence of protein degradation. Alum und LLOMe, on the other hand, degraded inflammatory proteins (including caspase-1), inhibited NALP3 signaling and led to necrotic death [157]. Interestingly, lysosomal degradation in cells treated with NALP3 inducers occurred *after* induction of cell death, further eroding support for the theory that lysosomal cathepsins are the activators of the inflammasome. Finally, the recent discovery of a caspase-1—independent, cathepsin-dependent pathway for the production of mature IL-1 [159] suggests that conclusions about the role of the inflammasome in cellular responses that are based on the measurement of IL-1 release will have to be re-evaluated. In particular, the role of alum-induced calcium mobilization in NLRP3 inflammasome activation may have to be assessed further [160].

While the contradictions generated by recent studies into alum’s mechanism of adjuvanticity may be frustrating, they have provided novel insights into cellular pathways and mechanisms, such as demonstrating the potential usefulness of agents that destabilize lysosomes (e.g., LLOMe) as adjuvants, or linking lysosome-mediated necrotic death to a Th2-bias of the subsequent adaptive response.

## 6. Beyond Macrophages and Dendritic Cells: Targeting Other Contributors to Vaccine-Induced Immunity by Specialized Adjuvants

Professional APCs, in particular DCs, are mainly cited as the target of vaccine adjuvants. These cells express a variety of innate immune receptors and are capable of activating naïve T cells, an essential feature for a vaccine containing antigens, which the vaccinee had not previously encountered. Interestingly, alum as well as MF59, two prototypic adjuvants, do not directly activate DC *in vitro* [161]. Bystander recruitment and activation of DC by other cells of the innate immune system likely lead to (or at least significantly contribute to) DC activation. These activated bystander cells can influence and shape the character of the inflammatory response, which subsequently determines the type of the lymphocyte profile (such as Th1, Th2, or Th17). There is also evidence that adjuvanting antigen by adding complement factors (C3d, C4b, C5), and thus targeting the formulation to complement receptor (CR)-expressing cells (such as B cells), greatly increases immunogenicity by crosslinking the surface immunoglobulins on B cells with the CR. The resulting high-avidity, long-lasting antibody responses often outperform responses induced by antigen emulsified in potent adjuvants (reviewed [12]).

Observations such as these justify the search for compounds that selectively activate other leukocyte subpopulations to potentially control the immune response and/or the safety profile of the vaccine. This area of adjuvant research is still in its infancy, but it opens exciting new opportunities for novel vaccines and the ability to tweak antibody responses by tapping into the ability of innate immune cells such as natural killer (NK) and natural killer T cells (NKT) or neutrophils to provide T-independent B cell help (reviewed in [162]).

### 6.1. Adjuvants Targeting Natural Killer and Natural Killer T Cells

A number of adjuvants have been reported to depend on additional compartments of the immune system that are not only bystanders of the inflammatory response, but also active responders to—and direct targets of—the adjuvant. While the recruitment of the recently identified long-lived (memory) NKT cell populations as effectors of a protective immune response may be an approach that is still be many years away, actively recruiting additional populations of innate immune cells during the priming of an adaptive immune response will more closely mimic the response to an active infection and is predicted to result in more robust adaptive immunity. Adding adjuvants that stimulate NK and/or NKT cells to a vaccine may provide a second benefit, in addition to stimulating a cell population that assists in the activation of lymphocyte responses. NK and NKT cells also participate directly in the response against various pathogens and inducing short-lived activation may be highly beneficial for therapeutic vaccines. Alpha-galactosylceramide (α-GalCer), a glycolipid from a marine sponge, binds to the CD1d surface molecule and stimulates NKT cells [163], which have been implicated in the immune response against pre-erythrocytic stages of malaria [164]. NKT-cell stimulation can also assist in the activation of DCs and thus greatly influence subsequent adaptive immune responses. Therefore, CD1-binding glycolipids are currently being explored as vaccine adjuvants (reviewed in [165]), where they enhance not only vaccine-specific T cell responses (e.g., for influenza [166], providing cross-protection against other viral strains [167] or the irradiated sporozoite-based malaria vaccine [168]), but also antibody titers [169], taking on the role of helper (T) cells [170]. In a clinical trial of α-GalCer, the compound was loaded onto adoptively transferred DC, resulting in NKT activation and NKT as well as CD8^+^ T cell expansion [171].

### 6.2. Adjuvants Targeting Mast Cells

Mast cells are present in large numbers close to the surface of the skin and mucosal tissues. Here, they act as sentinels, which contribute to subsequent adaptive immune responses, as well as effector cells against pathogens. Their activation is commonly described in the context of allergic or anaphylactic responses, which explains the reluctance of researchers to explore their potential as targets of adjuvants. However, their ability to instruct lymphocyte responses, their abundance, and their accessibility make them attractive targets for novel adjuvants. Even before they became a focus of targeted activation, it was reported that mast cells respond to the injection of alum into muscle tissue and some cytokines induced as a consequence of this injection are only generated in the presence of mast cells. However, it was also noted that the absence of mast cells did not compromise immune responses by alum-adjuvanted vaccines [172]. Mast cell-specific, small molecule compounds have been explored as mucosal adjuvants, and the “histamine liberator” compound 48/80, which was first described more than half a century ago [173], was shown to be a potent, but safe, adjuvant for experimental intranasal vaccines against anthrax toxin and vaccinia virus [174]. The intranasal route is attractive for several reasons, including the needle-free delivery of the vaccine and the potential for inducing strong mucosal immunity. Moreover, this immunization route may prime lymphocytes to home to tissues that are the natural entry points for various pathogens. Such imprinting of the adaptive response to circulate/reside in mucosal tissues has been a challenge for many intramuscularly-delivered vaccines.

## 7. Beyond Adjuvants: How to Properly Deliver Adjuvanted Vaccines

Whether or not a certain vaccine formulation will ever advance into clinical trials depends greatly on its reactogenicity and the anticipated safety profile. Like any medical procedure, vaccination is associated with a certain level of risk. The result of the risk-benefit analysis is overwhelmingly in favor of vaccines, but nevertheless, safety expectations, particularly for preventive vaccines and those given to children, are extremely high. Vaccination is associated with two types of safety concerns: (1) common, but relatively mild local reactions that occur predominantly shortly after vaccination and disappear quickly (most frequently pain, swelling, and reddening at the injection site and—less frequently—malaise, myalgia, fever). Mild side effects are often seen as a sign that the vaccine has induced an immune response and are more an annoyance to the vaccinee rather than an actual concern. (2) Serious adverse events are very rare and there are only a few examples of life-threatening or debilitating adverse events. Even though such responses are difficult to predict and uncommon, they significantly impact public opinion about vaccines and frequently result in the ill-guided decision to choose the (high) risk of a potentially life-threatening disease over the statistically negligible risk of an adverse vaccination event. 

Apart from reactogenicity and safety, there are many other factors that affect the immunogenicity and efficacy of a vaccine. The following are examples of such factors that should be considered when testing/optimizing vaccine formulations: 

### 7.1. Ensuring the Presence of Specific Immune Cells at the Pathogen’s Point of Entry

The routing of immune cells to specific tissues, where pathogen entry occurs or which are the main target of the pathogen (homing), is an important factor that affects the intensity and quality of an immune response and, thus, ultimately its protective efficacy. This process can be affected by the vaccine mainly by using different immunization routes and delivery systems. Most human vaccines are delivered by intramuscular immunization, inducing systemic immunity but not necessarily good, mucosal immunity. One notable exception is replication-deficient alphaviral particles, used as adjuvants and co-injected intramuscularly with recombinant vaccines [26]. These virus-derived particles have been shown to induce strong mucosal immune responses, together with systemic immune responses, thus providing two layers of immune protection. Many pathogens enter their mammalian host through mucosal routes and, therefore, strong mucosal immunity would be highly desirable. Mucosal vaccine delivery holds the promise of inducing both mucosal and systemic immunity. Due to the ease and lack of pain at the time of immunization, it may be a route that is more acceptable to many vaccine recipients, thus leading to higher compliance with vaccination requirements and a higher success rate of vaccination campaigns. 

### 7.2. Mucosal Vaccination—Why Not?

The effectiveness of mucosal vaccines has been demonstrated and influenza vaccination is a prime example. A comparison of the inactivated injectable *vs.* intranasally—delivered live attenuated influenza vaccine shows better efficacy of the latter [175]. From a practical standpoint, however, the development of mucosal vaccines faces several technical challenges: (1) The effects and the efficacy of adjuvants depends on the target site and many adjuvants that are suitable for needle/syringe based delivery to e.g.*,* muscle or skin are not suited for mucosal delivery to sites with significantly different immune cells expressing a different array of PRRs; (2) intranasally delivered formulations have to be tested in animals which have a nasal architecture similar to that of humans such as rabbits [176], making these studies more expensive. Rabbits are still not an ideal model since, for example, the olfactory epithelium occupies a significant portion of the nasal cavity, which is comparable to the situation found in rats, mice and dogs. In contrast, primates (including man) have relatively little olfactory epithelium [176]. Formulations need to be optimized for the mucosal route—for intranasal vaccination, strong mucoadhesion assures delivery to the nasal and not pulmonary mucosa, while in the case of intestinal vaccines, the inoculum has to become bioavailable at the correct anatomical site and withstand the passage through the stomach and upper intestinal tract; (3) PRR-ligands which provide good adjuvanticity when added to intramuscularly injected vaccines may not be useful as a mucosal adjuvant. While RNA-based PRR ligands such as poly(I:C) (TLR3 agonist) [177] or RNA from replication-deficient Sendai virus (RIG-I agonist) [178] can promote the induction of adaptive immune responses from a mucosal site, “legacy adjuvants” such as alum or Freund’s adjuvant (or more modern formulations such as MF59) are entirely unsuitable for this route; and (4) regardless of the anatomical site, mucosal vaccines, too, are subject to safety concerns. Using strong adjuvants for intestinal vaccines carries the risk of aggravating inflammatory diseases such as inflammatory bowel disease (IBD), Crohn’s disease, or celiac disease, conditions that are on the rise in developed countries. Enthusiasm for intranasal delivery of vaccines was drastically dampened by the increased risk of Bell’s palsy [179] following intranasal flu vaccination with the Nasalfu^®^ vaccine. Whether or not the mucosal adjuvant used for this vaccine (inactivated *E.coli* heat-labile toxin) was the culprit subsequently became the subject of a spirited discussion [180]. Novel types of adjuvants for mucosal delivery, such as nanoemulsions for intranasal vaccines [181], safely induce strong Th1 and Th17 immunity [182] without significant inflammation thus potentially eliminating a major concern for intranasal adjuvants.

### 7.3. Altering Immune Responses through Vaccination Regimens

Although undesirable, most vaccines require multiple immunizations before providing a significant level of protection from infection. The seasonal influenza vaccine may seem to be an exception, but previous exposures to cross-reactive and/or conserved influenza antigens through infection or vaccination provide a certain level of priming [183], and vaccine-induced protection only needs to last for one influenza season. Vaccines against complex pathogens, which counteract immune recognition by expressing weakly immunogenic antigens, require (1) multiple immunizations to establish immunity, and (2) likely regular booster immunizations to maintain protection. An example is the currently most advanced malaria vaccine candidate, RTS,S [184]. Although the initial immunization regimen involved three injections of the vaccine formulated with the potent AS01 adjuvant, which contains two separate adjuvants, MPL and QS21, protection significantly declined over the 4-year period following vaccination. 

Primarily for convenience, vaccination regimens are traditionally homologous whereby the exact same vaccine formulation is used for the prime and boost. Significant insights into clinical heterologous prime-boost regimens have been made in the HIV field [185] where homologous regimens have resulted in high-profile failures, such as the use of adenoviral vector serotype 5. DNA vaccine-induced responses can be boosted with “strong” vaccines without risking the induction of vector-neutralizing antibodies or reactogenicity following the repeated use of an adjuvanted vaccine. Considering the drastic increase in variables when using a heterologous regimen (such as choice of vaccine platforms to be combined, number of immunizations with one platform *vs.* the other), it can be expected that this already very promising approach [186] will continue to be refined and improved. However, the more complex immunization regimen will make this approach only attractive for diseases against which no effective vaccine is available yet. The potential benefit of changing vaccine formulations between prime and boost is not only limited to the vaccine platform, but can be extended to the choice of adjuvant; the benefits of changing adjuvants between prime and boost or using adjuvant-free formulations for the boost (to minimize or avoid reactogenicity) are only beginning to be explored.

### 7.4. Altering Immune Responses through Vaccination Intervals

Currently used intervals between immunizations with licensed vaccines are not necessarily optimal, but are sometimes modified for entirely non-scientific reasons: Many pediatric vaccines are squeezed into the same schedule in order to minimize office visits. This maximizes compliance with vaccination requirements. Vaccination regimens for preclinical models of vaccines traditionally involve intervals between injections that are selected based on convenience and published reports of vaccine studies using unrelated antigens and adjuvants. Longer intervals between immunizations are unpopular due to the higher costs associated with the longer duration of the experiment. Several studies have demonstrated the benefits of prolonging intervals between immunizations. In malaria, a preclinical study using plasmid DNA encoding the CSP antigen demonstrated that the parameter that most strongly affected the intensity, quality, and isotype of the vaccine-induced antibody response, as well as protection, was the interval [187]. The CSP-based clinical malaria vaccine RTS,S vaccine provides less than 50% protection in mosquito-bite challenged volunteers when using short (0/4/12 weeks or 0/7/28 days) vaccination intervals (first reported in [188]). However, by simply changing the interval to 0/4/28 weeks, efficacy increased to 80% even though the last vaccine dose was significantly decreased [189]. Shortened immunization intervals carry a frequently ignored, but significant, risk: After an initial antigen exposure it takes several weeks to generate memory T cells with high proliferative potential (reviewed in [190]). Repeated boosting can drive T cells towards terminal differentiation [191], risking the depletion of the antigen-specific central memory cell pool [189]. This might be acceptable in the case of therapeutic cancer. For example, in an animal model of melanoma, multiple weekly immunizations were necessary to break tolerance against tumor-associated self-antigens and generated effective anti-tumor immunity [192,193]. In contrast, the objective of a vaccine against an infectious disease in most cases is long-term protective immunity. Similarly to T cells, B cells, too, require time to mature after antigen exposure which may take several months [194]. 

## 8. Off-the-Shelf Vaccines Are Not for Everyone: Using Adjuvants for Targeted Solutions

The heterogeneity of human vaccine recipients represents a significant challenge for designing safe and efficacious vaccination strategies. Off-the-shelf vaccines are based on the assumption that the immune responses they induce are somewhat homogenous in the general population. The challenge to achieve full immune protection by vaccination is best illustrated by the fact that the licensed HBV vaccine—for mostly unknown reasons—fails to induce lasting seroprotection in 5%–10% of immune-competent vaccinees. Such problems may be overcome by using different adjuvants either for the general population (although a safe and mostly effective vaccine will unlikely be replaced by a novel formulation) or for the non-responders (who are easily identified after a first unsuccessful immunization). In the case of HBV, a CpG-adjuvanted vaccine has been shown to outperform the currently used alum-adjuvanted vaccine [195]. 

### 8.1. Heterogeneity of the Adaptive Immune Response in the Human Population

HLA diversity results in the individual selection of immunodominant T cell epitopes with a potentially significant impact on the quality of the response. The selective recognition of pathogen-derived epitopes which—either by chance or purposely (molecular mimicry)—cross-react with host epitopes may lead to the induction of autoimmunity by a vaccine in a subpopulation of vaccine recipients. This process will be affected by the choice of adjuvant. The induction of narcolepsy in a small number of children following vaccination with the pandemic H1N1 vaccine has been hypothesized to represent a situation where cross-reactive viral epitopes became immunodominant (triggering autoimmunity). This only occurred (a) in the presence of the AS03 adjuvant (AS03 in combination with a pandemic influenza vaccine was licensed by the FDA in November 2013, representing only the second FDA approved vaccine with an adjuvant other than alum [196]), but not MF59 [197], a remarkably similar adjuvant formulation) and in individuals expressing (b) a particular HLA (HLA DQB1*06:02) and/or (c) TCRα locus [198]. A candidate self-antigen was recently identified [199]. This experience is a reminder that adjuvant selection in humans is not determined solely by efficacy and general safety, but needs to take into account rare (and sometimes unexpected) adverse events, which explains the reluctance of regulatory agencies to approve novel adjuvants.

### 8.2. Heterogeneity of the Innate Immune Response in the Human Population

Responses to adjuvanted vaccines are also significantly affected by polymorphisms in innate immune receptors (e.g., TLR [200]) and/or other immune factors [200,201] and have not only been linked to autoimmunity (reviewed in [202]) but also been blamed for the failure of certain vaccines [203]. Cytokine expression levels are controlled by polymorphisms and for example in the case of IL-10, these polymorphisms have been shown to correlate with disease susceptibility (and likely also responsiveness to vaccines) when modeled in humanized mice [204]. Although the obvious solution to this problem—personalized vaccines—is not feasible on a large scale, information about common polymorphisms in crucial innate immune genes in a particular population can be used to select adjuvants which do not depend on the affected receptor or factor.

### 8.3. Heterogeneity Based on Age and Immune Status of the Human Population

Significant differences exist between immune responses of adults and newborns, the latter being the prime target for preventive vaccines. Furthermore, an ever increasing number of immune-compromised individuals require vaccines, but may not respond well to those currently available. These include the elderly (with their increased need for protective immunity against influenza but impairment of immunity due to immune-senescence [205,206]), those with chronic illness (HIV, Hepatitis virus, diabetes) or on immunosuppressive drugs, or obese individuals [207,208]. Different vaccine formulations employing stronger adjuvants may be needed for these populations. In the case of newborns, a better understanding is needed of the immune components that are not fully developed early in life, such as the failure of neonatal Tfh to properly support functional germinal center responses and thus antibody production [209]. Insights into the differential distribution of TLRs or the difference in their responsiveness to ligands compared to their adult counterparts [206] can guide the selection of adjuvants specifically for neonatal vaccines [210].

### 8.4. Heterogeneity of the Global Population

A number of vaccines which work well in industrialized countries (or which were successfully tested in healthy volunteers from developed countries) failed when given to populations in developing countries [211]. Numerous reasons have been identified, including pre-existing immunity to other strains of the same pathogen in the case of a malaria vaccine [212], and poor nutritional status, or co-infections with pathogens such as intestinal worms which bias immune responses towards a Th2-profile. The finding that several of these factors can be overcome by a strong vaccine such as the licensed measles vaccine suggests that vaccines for these recipients may simply need to be reformulated with sufficiently strong immune stimulators to provide the same benefit [213].

## 9. Conclusions

The need for new adjuvants will increase as the need for new and more effective vaccines continues to grow. Novel vaccines are needed for diseases for which none exist currently and for diseases which apparently cannot be tamed by conventional approaches such as attenuated pathogen or recombinant vaccines. But new vaccines are also needed for diseases that are being insufficiently controlled by currently available vaccines. Included in the latter category are all licensed vaccines that require multiple boosters to achieve protection. Their “poor” efficacy is due to insufficient patient compliance with vaccination regimens or they are vaccines which do not provide seroconversion in all vaccinees. Even the seasonal flu vaccine would greatly benefit from adjuvant for dose sparing and efficacy in the elderly and other special populations. Because of ever increasing safety concerns, there are significant reservations when it comes to the inclusion of new adjuvants in vaccines. 

### 9.1. Are We There Yet?

The path from discovery of a novel adjuvant to clinical application is long, difficult and expensive [79]. The best example is MPL (MLA) in combination with alum (AS04), approved for used with the HPV vaccine Cervarix^®^ [214]. Lipopolysaccharide was first described as a potential adjuvant in 1955 [215]; nevertheless, it took more than half a century for an adjuvant based on this potent PAMP to become part of a vaccine licensed in the U.S. 

### 9.2. Current Trends in Adjuvant Research

#### 9.2.1. Heterologous Prime-Boost

Most vaccination regimens involve the use of exactly the same vaccine formulation for priming and each booster immunization. While heterologous prime-boost regimens involving different vaccine platforms (e.g., priming with DNA and boosting with a recombinant vector) have shown great promise, for example, in malaria [216] and HIV [217]; another heterologous prime-boost approach has recently received more attention: It may be advantageous to prime with an exogenously adjuvanted vaccine, while using a non-adjuvanted vaccine for the boost. The antigen in this case can be purified protein, as shown in a study using replication-deficient viral particles as adjuvants for the prime only [26]. Using adjuvant only for the first immunization can reduce the number of vaccination-related adverse events which tend to be stronger after a boost with an adjuvanted vaccine, This has, for example, been reported for the RTS,S malaria vaccine [189], suggesting that antigen-specific immune responses may have impacted innate immunity [41]. In the context of influenza vaccines, using prime-boost regimens with different strains of pandemic influenza vaccines in an attempt to broaden cross-strain protection indicated that inclusion of an adjuvant was required for priming, but not boosting. Priming without adjuvant was reported to prevent efficient boosting, representing a case of original antigenic sin [218].

#### 9.2.2. Combination Adjuvants and the Future of Alum

The high comfort levels with alum, and the fact that billions of doses of alum have been deployed with an incredibly small number of adverse events, make this a highly attractive adjuvant which will continue to be used. However, alum as the sole adjuvant is inadequate for many vaccines. Instead, alum has been “re-discovered” as a co-adjuvant/carrier and is used in combination with (other) immunostimulatory compounds. The regulatory path for such combination adjuvants has been illuminated by the licensure of an AS04-adjuvanted vaccine. Alum or other particulate adjuvants/carriers (such as PLGA particles) may not only provide synergistic immunostimulatory signals but also deliver e.g., TLR agonist to DC, and slow their release, thus reducing reactogenicity. 

#### 9.2.3. Overall Combination Adjuvants

Combining multiple adjuvants such as different TLR agonists may allow tweaking of the immune response to obtain higher quality, although not necessarily higher quantity (e.g., higher efficacy was shown with three TLR-agonists in combination compared to only using two, although antigen-specific T cell numbers were the same [219]). While the benefit of stimulating multiple PRR in the form of enhanced immune responses is widely accepted and has been shown to be a major factor for making the Yellow Fever Vaccine one of the most effective vaccines available [82], interactions between major PRR pathways to identify additive, synergistic or inhibitory interactions have not been studied very well, with only one publication describing a systematic analysis of combinations of ligands for PRRs [142]. In addition, PRR-agonists have been combined with other adjuvants resulting in a synergistic enhancement of adaptive immunity (e.g., poly(I:C) plus ISCOM [99], or adding both a TLR9 agonist and the DC-expanding factor Fms-like tyrosin kinase 3 (Flt3)-ligand to a heterologous prime (DNA plasmid)-boost (viral vector) regimen [220]. Many other combinations of adjuvants are currently being explored with “adjuvants” also including recombinant or plasmid-encoded chemokines and cytokines.

#### 9.2.4. Comparative Adjuvant Research

A significant number of studies involving vaccine adjuvants are based on a single immunopotentiator. The usefulness of a particular adjuvant depends on a variety of factors including the antigenic component. Considering the significant gaps in understanding (1) immune correlates of protection against many pathogens and (2) the mechanism of action of many adjuvants, combining one select antigen/vaccine with one select adjuvant is rather random and associated with a significant risk of failure. For example, a transmission-blocking vaccine for malaria based on the Pfs40 antigen failed in five different mouse strains when adjuvanted with Freund’s adjuvant. While this formulation is often used as a “gold standard,” adjuvanting the vaccine with MPL and TDM induced the expected antibody response [221]. Increasingly, multiple adjuvants are compared side-by-side, using the same antigen/vaccine to determine the “best fit” for a particular antigen. Such studies are also helpful in providing novel insights into immune mechanisms of protection by allowing a comparison of protective *vs.* non-protective immune profiles obtained with vaccines containing the same antigenic components (reviewed in [41]). 

#### 9.2.5. Novel Targets

Until a decade ago, rational adjuvant research was dominated by TLR agonists. The characterization of additional PRR has significantly broadened the field and RIG-I has attracted a lot of attention as a target for adjuvants [222], using formulations based on the natural ligand, RNA (dsRNA [178,223]), or on replication-defective particle [224]. Although other RNA-based adjuvants are already at the stage of advanced development, namely synthetic double-stranded (ds) RNA such as poly(I:C) targeting TLR3, the response profiles of the different types of RNA-sensors (TLR3, RLRs, PKR) are significantly different. These insights justify the parallel development of multiple agonists for these receptors as vaccine adjuvants. Novel innate immune receptors continue to be discovered as well as targets for adjuvants directly expressed on lymphocytes (e.g., complement receptor [12]) and therefore, the spectrum of candidate adjuvants can be expected to drastically expand in the coming years.

#### 9.2.6. Mechanism of Action

For decades, adjuvants were used without a real understanding of how they provided adjuvanticity. The enduring dogma that alum works through providing a depot effect was only recently debunked. The relatively recent focus on C-type lectins as targets of novel adjuvants has provided an unexpected insight into the mechanism of another “legacy adjuvant”: mycobacterial glycolipid trehalose dimycolate (cord factor/ trehalose-6,6'-dimycolate) is one of the active components of complete Freund’s adjuvant and acts through mincle (Clec4e), resulting in Th1 and Th17 induction [129,225]. Discoveries of mechanisms of action of adjuvants are not only fueled by our improving understanding of innate immune pathways but are in turn themselves drivers of progress in innate immunity research. These insights will assist in the rational design of new, increasingly safe and progressively more targeted adjuvants. 
